# Establishing the Melbourne injecting drug user cohort study (MIX): rationale, methods, and baseline and twelve-month follow-up results

**DOI:** 10.1186/1477-7517-10-11

**Published:** 2013-06-21

**Authors:** Danielle Horyniak, Peter Higgs, Rebecca Jenkinson, Louisa Degenhardt, Mark Stoové, Thomas Kerr, Matthew Hickman, Campbell Aitken, Paul Dietze

**Affiliations:** 1 Centre for Population Health, Burnet Institute, 85 Commercial Rd, Melbourne, VIC 3004, Australia; 2 Department of Epidemiology and Preventive Medicine, Monash University, 99 Commercial Rd, Melbourne, VIC 3004, Australia; 3 Kirby Institute, University of New South Wales, Corner Boundary and West Streets, Darlinghurst, NSW 3020, Australia; 4 National Drug Research Institute, Curtin University, 54-62 Gertrude St, Fitzroy, VIC 3065, Australia; 5 Centre for Health Policy, Programs and Economics, School of Population Health, University of Melbourne, Level 5, 207 Bouverie St, Melbourne, VIC 3010, Australia; 6 National Drug and Alcohol Research Centre, University of New South Wales, 22-32 King St, Randwick, NSW 2031, Australia; 7 Urban Health Research Initiative, British Columbia Centre for Excellence in HIV/AIDS, Vancouver, BC V6Z 1Y6, Canada; 8 Department of Medicine, University of British Columbia, Vancouver, BC V6T 1Z4, Canada; 9 School of Social & Community Medicine, University of Bristol, Canynge Hall, Bristol BS8 2PS, United Kingdom

**Keywords:** Injecting drug use, Cohort, Longitudinal studies, Australia

## Abstract

**Background:**

Cohort studies provide an excellent opportunity to monitor changes in behaviour and disease transmission over time. In Australia, cohort studies of people who inject drugs (PWID) have generally focused on older, in-treatment injectors, with only limited outcome measure data collected. In this study we specifically sought to recruit a sample of younger, largely out-of-treatment PWID, in order to study the trajectories of their drug use over time.

**Methods:**

Respondent driven sampling, traditional snowball sampling and street outreach methods were used to recruit heroin and amphetamine injectors from one outer-urban and two inner-urban regions of Melbourne, Australia. Information was collected on participants’ demographic and social characteristics, drug use characteristics, drug market access patterns, health and social functioning, and health service utilisation. Participants are followed-up on an annual basis.

**Results:**

688 PWID were recruited into the study. At baseline, the median age of participants was 27.6 years (IQR: 24.4 years – 29.6 years) and two-thirds (67%) were male. Participants reported injecting for a median of 10.2 years (range: 1.5 months – 21.2 years), with 11% having injected for three years or less. Limited education, unemployment and previous incarceration were common. The majority of participants (82%) reported recent heroin injection, and one third reported being enrolled in Opioid Substitution Therapy (OST) at recruitment. At 12 months follow-up 458 participants (71% of eligible participants) were retained in the study. There were few differences in demographic and drug-use characteristics of those lost to follow-up compared with those retained in the study, with attrition significantly associated with recruitment at an inner-urban location, male gender, and providing incomplete contact information at baseline.

**Conclusions:**

Our efforts to recruit a sample of largely out-of-treatment PWID were limited by drug market characteristics at the time, where fluctuating heroin availability has led to large numbers of PWID accessing low-threshold OST. Nevertheless, this study of Australian injectors will provide valuable data on the natural history of drug use, along with risk and protective factors for adverse health outcomes associated with injecting drug use. Comprehensive follow-up procedures have led to good participant retention and limited attrition bias.

## Background

People who inject drugs (PWID) are exposed to blood-borne virus (BBV) infections [1,2], injecting-related injuries [3,4] and risk of overdose [5-7], and experience greater levels of both physical and mental impairment compared with the general population [8-14]. Meta-analysis of cohort studies has shown that PWID have a greatly increased risk of premature death, attributable to both AIDS and non AIDS-related causes [15], with mortality among opiate injectors estimated to be approximately 19 times higher than the general population [16]. Additionally, injecting drug use is associated with a range of social and economic harms [17-21].

Our ability to respond to the significant morbidity and mortality associated with injecting drug use is limited by our lack of understanding of the complex ways in which drug-related harms are produced, and the ways in which interventional efforts can be optimised. Most Australian and much international research among this population has been cross-sectional, which captures only a single time point and cannot explore how patterns of risk behaviour, and subsequent health outcomes may change during a person’s injecting career.

Cohort studies provide a unique opportunity to measure changes in behavior and disease transmission over time. They can, however, be difficult studies to conduct; they require sufficient funding to facilitate follow-up over time [22], and are subject to cohort effects, as well as selection bias if they experience high levels of attrition, particularly if loss to follow-up is associated with important participant characteristics [23,24]. Additionally, controlling for confounding when assessing relationships between behaviour and disease transmission can prove challenging [25,26].

Cohort studies involving PWID have proven especially difficult; although studies have achieved follow-up rates of 68-80%, attrition is often associated with factors such as homelessness, incarceration and early death [27-33]. While a number of successful PWID cohorts are ongoing in the USA and Canada, in Australia such studies have been relatively rare. In Australia, longitudinal studies among PWID have been conducted among in-treatment samples [34], which comprise mainly long-term injectors who are either injecting infrequently or not at all, and thus may not provide accurate information about the prevalence and incidence of risk behaviour and disease. When community-based cohorts have been conducted, they have been limited by short duration of follow-up [35,36]. Outcomes measured in these studies have primarily focused on either hepatitis C incidence or drug treatment outcomes, with limited data collected on health outcomes more broadly [34-37]. Further, most studies involving PWID in Australia are generally focused on an older sample of PWID who initiated and became entrenched in injecting drug use in the mid-late 1990s, a period that was characterised by the ready availability of heroin [38] - markedly different to the drug market characteristics of today. It is not clear whether patterns of drug use and related risk behaviour among this older cohort is reflective of newer, younger injectors. For these reasons, we need long-term studies of Australian PWID that include those people who have commenced injecting more recently and continue to regularly inject drugs in contemporary drug market conditions.

The Melbourne Injecting Drug User Cohort Study (MIX) was designed to explore the natural history of injecting drug use, as well as to identify risk and protective factors for adverse health outcomes and health service utilisation among PWID. We aimed to recruit a large sample of young, out-of-treatment PWID, with equal numbers preferring heroin or methamphetamine as their drug of choice. In this paper we report on methods used to recruit and retain MIX participants, describe the cohort’s baseline characteristics, and explore factors associated with attrition at 12 months follow-up.

## Methods

### Setting

**Figure 1:**
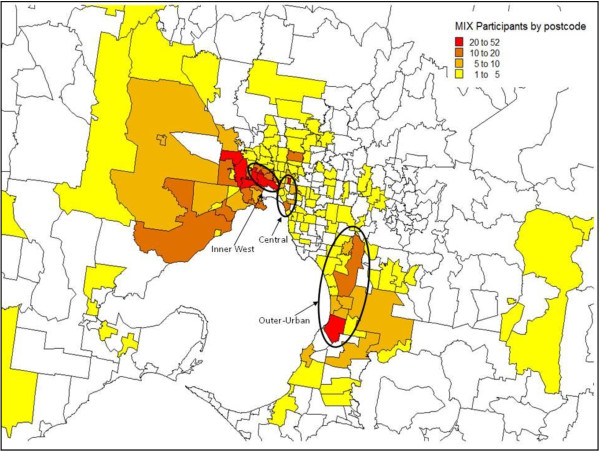
Geographic location of recruitment sites, and distribution of participants by postcode of residence at baseline.

The study was conducted in Melbourne, the second largest city in Australia (population ~4 million (2009)) and capital city of the state of Victoria [39]. Baseline recruitment was conducted between November 2008 and March 2010, across one outer-urban and two inner-urban (Inner-West and Central) areas of Melbourne where illicit drug markets had been identified through previous studies and/or where primary needle and syringe exchange programs (NSPs) were located (Figure 1).

### Eligibility criteria

Individuals were eligible for the study if they: (1) reported being aged between 18 and 30 years old; (2) had injected either heroin or methamphetamine at least six times over the previous six months; (3) were currently residing in Melbourne; (4) were willing to provide detailed contact information including their full name, residential address and telephone number; and (5) were able and willing to provide a valid Medicare card number, to be used, along with other personal details, for data linkage (Medicare is Australia’s universal health-care system which provides access to free or subsidised medical and allied health services; the Medicare number is unique for each individual listed on the system).

A sixth criterion, ‘not currently being prescribed Opioid Substitution Therapy (OST)’ was withdrawn three months into the study due to the high number of otherwise eligible participants who were being excluded (only 31 participants were enrolled into the study during this time). This decision was made in light of the drug market situation in Melbourne at the time, where fluctuating heroin availability and purity led to ever-increasing numbers of PWID accessing low-threshold OST and cycling in and out of treatment regularly [38,40,41].

Amendments were also made to the selection criterion regarding age, as it came to light that the PWID population in Melbourne is ageing, while uptake of injection is decreasing [42], making it difficult to recruit younger PWID. As such PWID who were slightly older than the target age range, but were not on OST, were also included in the study. The financial and time constraints of the longitudinal study design were also a factor in these decisions.

### Pilot interviews

Thirty-two pilot interviews were conducted between March and August 2008 to identify any ambiguities or other problems within the questionnaire. Pilot participants were PWID who were previously known to researchers, and who met the study eligibility criteria. Pilot interviews are not reported separately to baseline data in this report.

### Recruitment strategies

Participants were recruited using Respondent Driven Sampling (RDS), street outreach and snowball sampling, in order to maximise the number and diversity of participants recruited over a limited time period [43].

#### *Respondent driven sampling*

RDS is a modified chain-referral sampling technique used for the recruitment of hard-to-reach populations [44]. A small number of ‘seed’ participants are selected from the chosen population, and monetary incentives are used to facilitate recruitment of additional participants through seeds’ social networks. Weighted analysis based on social network sizes is conducted to adjust for the bias that is generally associated with chain-referral methods [44,45].

Up to five PWID from each recruitment site who were known to study researchers through participation in previous studies or through agency referral, and met the study eligibility criteria, acted as the seeds. Following interview, each seed received a set of uniquely numbered recruitment coupons and was invited to recruit a maximum of three peers into the study. The coupons directed interested parties to contact researchers via a free-call telephone number, in order to be screened for study eligibility. Once eligibility was confirmed, an appointment time was made to conduct the interview. Additional seeds were added as required to boost recruitment (on an ad hoc basis).

#### *Street outreach and snowball sampling*

A team of researchers regularly attended each of the recruitment locations. Eligible participants were recruited through word of mouth and flyers posted in relevant community agencies. PWID who met the eligibility criteria and were known to researchers through participation in previous studies were also actively recruited. These participants were then given the opportunity to invite their contacts to also participate in the study. Participants recruited through street outreach and snowball sampling also received RDS coupons to distribute to their peers. All participants who returned an RDS coupon are considered as having been recruited through the RDS arm of the study.

### Questionnaire design and administration

Interviewer-administered questionnaires were conducted using hand-held personal digital assistants (PDAs). Information was entered into a database constructed using Questionnaire Design System Versions 2.4-2.6 (NOVA Research Company, Maryland, USA).

To protect participant confidentiality, contact details and survey data were entered into two separate databases. Detailed contact information was recorded to enhance the likelihood of successful follow-up, including the participant’s full name, date of birth, alias or street name, residential address, land and mobile telephone numbers, and contact details for a nominated friend or relative who was likely to know the participant’s whereabouts during the study. Medicare number and RDS coupon details were also recorded in this database. A unique identifier was assigned to each participant using an algorithm based on the participant’s first name, surname and year of birth.

The study questionnaire covered four domains: demographic and social characteristics; drug use characteristics and drug market access; health and social functioning; and health service utilisation. Standardised and validated questionnaire items were used where appropriate. Details of selected variables collected are outlined in Table 1.

**Table 1:** Summary of variables collected at baseline interview

**Area of interest**	**Variables collected**	**Details**
Demographic and social characteristics	Gender	^1^Questions from the criminality section of the Opiate Treatment Index (OTI) were used to measure prevalence of property crime, violent crime, drug dealing and fraud in the past month
	Date of birth	
	Education status	
	Employment history	
	Income	
	Current living circumstances	
	Country of birth	
	Language spoken at home	
	Indigenous status	
	Criminal activity (including OTI^1^)	
	Incarceration history	
Dug use characteristics	Age at injecting initiation	^2^The Alcohol Use Disorders Identification Test (AUDIT) was developed by the World Health Organisation as a brief assessment tool to identify hazardous and harmful patterns of alcohol consumption, focussing primarily on symptoms occurring in the recent past
	Pattern of drug use at injecting initiation	
	Alcohol use (AUDIT^2^)	
	Drug use history	
	Current drug use	
	Drug market access and purchase characteristics	
	Drug treatment history	
	Social networks	
Health and social functioning	Height	^3^The Short-Form 8 (SF-8) assesses physical and mental health over the past month based on questions covering eight domains: physical functioning, role limitations due to physical health, bodily pain, general health perceptions, vitality, social functioning, role limitations due to emotional problems, and mental health.
	Weight	
	Chronic health conditions	
	Physical and mental health (SF-8^3^)	
	Quality of life (PWI^4^)	^4^The Personal Wellbeing Index (PWI) uses an 11-point Likert scale to measure quality of life according to eight domains: standard of living, health, achieving in life, relationships, safety, community-connectedness, future security, and spirituality/religion.
	BBV testing history and current status	
	Risk of BBV infection (BBV-TRAQ-SV^5^)	
	Drug overdose history	
		^5^The Blood Borne Virus Transmission Risk Assessment Questionnaire Short Version (BBV-TRAQ-SV) measures participation in high-risk practices for the transmission of blood-borne viruses. It consists of 15 items relating to needle and syringe contamination, other injecting equipment sharing, and second person contamination.
Health service utilisation	Type of services attended	
	(e.g. hospital, GP, PWID PHC clinic)	
	Frequency of service attendance	
	Reasons for attendance (drug-related, other)	
	Costs incurred for attendance	

Eligibility screening and interviews were conducted on-site either in a public space (e.g. park, outdoor cafe) or in a mobile study van, with interviews taking 39 minutes on average to complete (SD: 18 minutes). Participants were reimbursed AU$30 (US$19.83 in November 2008) for their time and out-of-pocket expenses in accordance with accepted practice [46], and an additional AU$10 for each coupon returned which resulted in an eligible interview.

### Follow-up procedures

Ideally, participants will be followed up annually for a minimum of four years (incorporating completion of a structured interview, as well as the collection of a blood sample for BBV testing). Given the anticipated difficulty in retaining participants we employed a variety of strategies to maintain contact with participants between interviews.

In addition to the extensive contact information collected at baseline, participants received a follow-up card noting the approximate date of their next interview and listing a free-call telephone number to contact researchers and update their details as required. Field-based researchers maintained contact with participants they encountered in the field, and updated contact details when possible.

Two to four weeks prior to their scheduled follow-up date researchers attempted to contact participants, initially via telephone (using both voice calling and text messaging). If telephone contact was unsuccessful researchers posted a letter to the participant’s home address or attempted contact through their nominated friend or relative. Field-based researchers actively sought out participants who were due for follow-up, and systematically recorded information received through their networks about a participant’s whereabouts (e.g. if they had been incarcerated). Telephone interviews were conducted with participants who were no longer residing in Melbourne if valid contact details were available.

In order to maximise the number of participants completing each follow-up interview, interviews could be conducted up to two months prior to the scheduled follow-up date if opportunistic contact was made. There was no end-point at which participants became ineligible to complete an interview, however, to avoid overlap in referent time periods, at least six months must have elapsed between interviews.

The follow-up interview was conducted using the same procedures as the baseline interview, with minor changes to the questionnaire to reduce repetition and incorporate prospectively occurring events. Participants were again reimbursed AU$30 per interview. Receipt of further study funding facilitated the collection of venous blood samples, to be tested for HIV, hepatitis B and hepatitis C infection. Participants who agree to provide a blood sample at each follow-up interview receive an additional AU$10 for the extra time and inconvenience involved.

### Staff training

Study staff received extensive training in field-based data collection, including the use of PDAs, administration of the questionnaire and adherence to standard operating procedures for field-based researchers, as well as completing accredited training courses in phlebotomy and BBV pre-and-post-test counselling.

### Ethics approval

The study was approved by the Victorian Department of Human Services (now Department of Health) and Monash University Human Research Ethics Committees. Written informed consent, including consent to access Medicare information, was obtained from all participants.

### Analysis and reporting

We conducted analyses to explore variations in participant socio-demographic characteristics, patterns of drug use and health status by recruitment site using the chi-square test for categorical variables, Wilcoxon rank-sum test for non-parametric continuous variables and the Kruskal-Wallis test for non-parametric continuous variables across more than two groups. Multivariable logistic regression was conducted to identify independent correlates of attrition at 12-months follow-up. Analyses were conducted using Stata Version 11.1 (Statacorp LP, Texas, USA), with a significance level of p<0.05. Missing data are not reported.

This manuscript has been prepared in accordance with the Strengthening the Reporting of Observational Studies in Epidemiology (STROBE) Statement [47].

## Results

### Baseline characteristics

Six hundred and ninety-four PWID were recruited into the study, but due to a technical error, baseline data for six participants were lost, resulting in a final sample of 688 participants. The median age of participants was 27.6 years (IQR: 24.4 years – 29.6 years). Participants were predominantly male (67%) and had been injecting drugs for a median of 10.2 years (range: 1.5 months – 21.2 years), with 11% of participants reporting injecting for three years or less (n=76). The majority of participants had not completed high school (80%), were unemployed (86%) and were dependent on government benefits as their main source of income (86%). One hundred and thirty-one participants (19%) reported being homeless or living in unstable accommodation such as boarding houses at the time of interview. The vast majority of participants reported injecting heroin during the month prior to recruitment (82%, n=563). Of the remaining participants, 27% reported only recent amphetamine injection (n=34), 48% reported injecting neither heroin nor amphetamine but other drugs, predominantly pharmaceutical opiates (n=60) and 25% had abstained from drug injection in the past month (n=31). One third of participants (35%) were prescribed OST at the time of interview, with those out-of-treatment significantly younger (median age: 27.3 years vs. 28.2 years; p=0.025), than those in treatment.

One third of participants were recruited through RDS (36%, n=246), with RDS-recruited participants generally similar to those recruited through street outreach and snowball sampling. Fifty-three per cent of participants (n=361) were recruited from Melbourne’s Inner West, 26% from Central Melbourne (n=177), and 22% from the Outer-urban site (n=150). Participants generally resided in close proximity to recruitment sites (Figure 1). Significant differences were detected in socio-demographic and drug use characteristics of participants across recruitment sites (Table 2). Participants from the Inner-West and Central sites were less likely to be born in Australia compared with those from the outer-urban site (76% and 77%, respectively vs. 93%), reflecting the significant South-East Asian and Horn of Africa migrant communities in these areas. Patterns of substance use varied across sites, with 40% of participants from each of the inner-urban sites reporting abstaining from alcohol consumption in the past month, compared with 23% of participants from the outer-urban site. Participants from the outer-urban site commenced injecting at a median age of 16 years (IQR: 14–18), slightly younger than other participants (median: 17, IQR: 15–20 in Inner-West, and 17, IQR: 15–19 in Central), and were significantly less likely to report heroin as their first drug injected (52% vs. 72% and 60% in Inner-West and Central respectively). At baseline, a smaller proportion of outer-urban participants reported recent heroin injection compared with those from other areas (50% vs. 94% (Inner-West) and 86% (Central)), with 11% injecting amphetamines only, and 32% injecting other drugs only. Frequency of recent heroin injection was lowest in the outer-urban site, where a greater proportion of participants reported being on OST at baseline (48% vs. 34% in Inner-West and 28% in Central). Patterns of recent attendance at PWID-specific primary health care (PHC) services, general practice (GP) clinics and hospital outpatient clinics were also significantly different across recruitment sites.

**Table 2:** Socio-demographic, drug use and health characteristics at baseline, by recruitment location

**Variable**	**Recruitment location**			***χ***^**2**^
	**Inner-West**	**Central**	**Outer-urban**	
	**N=361**	**N=177**	**N=150**	**p-value**
	**n (%)**	**n (%)**	**n (%)**	
**Recruitment method**				0.615
RDS	130 (36)	67 (38)	49 (33)	
Other	231 (64)	110 (62)	101 (67)	
**Demographic and social characteristics**				
**Sex**				0.920
Female	119 (33)	58 (33)	52 (35)	
Male	242 (67)	119 (67)	98 (65)	
**Age**				
Median (IQR)	27.4 (24.4-29.3)	28.0 (24.4-29.8)	27.8 (23.9-29.6)	0.579
**Aboriginal/Torres Strait Islander status**				0.534
Yes	22 (6)	12 (7)	6 (4)	
No	339 (94)	165 (93)	144 (96)	
**Country of birth**				<0.001
Australia	275 (76)	136 (77)	139 (93)	
Other	84 (24)	40 (23)	11 (7)	
**Main income source (last month)**				0.140
Wage or salary	28 (8)	15 (9)	15 (10)	
Government pension or benefits	308 (85)	150 (85)	131 (89)	
Other^1^	25 (7)	11 (6)	2 (1)	
**Employment status**				0.547
Not employed	311 (86)	154 (87)	125 (83)	
Employed	50 (14)	22 (13)	25 (17)	
**Education**				0.055
Did not complete year 10	118 (33)	49 (28)	64 (43)	
Completed year 10–11	169 (47)	85 (48)	63 (42)	
Completed high school or higher	74 (21)	43 (24)	23 (15)	
**Current accommodation type**				<0.001
Owner-occupied	97 (27)	27 (15)	30 (20)	
Private rental	103 (29)	41 (24)	48 (32)	
Public housing	104 (29)	54 (30)	49 (33)	
No stable accommodation	55 (15)	55 (31)	21 (14)	
**Incarceration history**				0.150
Never been in prison	145 (40)	76 (43)	55 (37)	
Incarcerated once	123 (34)	46 (26)	42 (28)	
Incarcerated two or more times	92 (26)	54 (31)	51 (35)	
**Recent arrest (last 12 months)**				0.299
Yes	201 (56)	86 (49)	82 (56)	
No	159 (44)	89 (51)	64 (44)	
**Drug use characteristics**				
**Age at first injection**				<0.001
Median (IQR)	17 (15–20)	17 (15–19)	16 (14–18)	
**Duration of injecting career (years)**				0.004
Median (range)	9.7 (<1-20.5)	10.2 (<1-21.2)	11.3 (1.1-21.2)	
**First drug injected**	261 (72)	106 (60)	78 (52)	<0.001
Heroin	91 (25)	63 (36)	59 (39)	
Amphetamines	4 (1)	5 (3)	1 (1)	
Other stimulant	3 (1)	2 (1)	10 (7)	
Other opiate	2 (1)	1 (1)	2 (1)	
Other				
**Drugs injected last month**				<0.001
Heroin only	172 (48)	85 (48)	22 (15)	
Heroin and other drugs	165 (46)	67 (38)	52 (35)	
Amphetamines only	9 (3)	8 (5)	17 (11)	
Other drugs only^2^	4 (1)	8 (5)	48 (32)	
Did not inject in last week	11 (3)	9 (5)	11 (7)	
**Frequency of heroin injection (last week)**				0.022
Median (range)	5 (1–60)	4 (1–50)	3 (1–28)	
**Frequency of methamphetamine injection (last week)**				0.047
Median (range)	2 (1–25)	2 (1–42)	2 (1–28)	
**Ever been on OST**				0.072
Yes	260 (72)	115 (65)	114 (77)	
No	99 (28)	61 (35)	35 (23)	
**Currently on OST**				0.001
Yes	121 (34)	50 (28)	71 (48)	
No	238 (66)	126 (72)	78 (52)	
**Frequency of alcohol use (last month)**				0.001
Never	146 (40)	69 (39)	34 (23)	
Once per week or less	119 (33)	48 (27)	51 (34)	
Two to three times per week	31 (9)	20 (11)	26 (18)	
Four or more times per week	65 (18)	39 (22)	38 (26)	
**Heroin overdose (lifetime)**				0.654
Yes	141 (39)	68 (39)	64 (43)	
No	219 (61)	107 (61)	84 (57)	
**Heroin overdose (last six months)**				0.799
Yes	36 (26)	18 (27)	14 (22)	
No	104 (74)	50 (74)	50 (78)	
**Health characteristics**				
**BBV status (self-reported)**^**3**^	n=324	n=162	n=143	
HCV positive	164 (51)	80 (49)	64 (45)	0.863
**Number of health services used (last month)**	n=352	n=172	n=148	
Median (range)	1 (0–7)	1 (0–9)	1 (0–5)	0.013
**Health services used (last month)**^**4**^				
Hospital Emergency Department	47 (13)	22 (12)	24 (16)	0.593
Hospital Inpatients	14 (4)	10 (6)	11 (7)	0.260
Hospital Outpatients	10 (3)	15 (9)	8 (5)	0.012
General Practice	200 (56)	95 (54)	105 (71)	0.003
PWID Primary Health Care Centre	55 (15)	41 (23)	22 (15)	0.047
Ambulance	25 (7)	15 (9)	14 (9)	0.620
Psychologist/psychiatrist	45 (13)	30 (17)	22 (15)	0.362
Other^5^	45 (13)	33 (19)	22 (15)	0.165

^1^ Includes criminal activity, sex work, being supported by spouse or family member, no current income.

^2^ Includes cocaine, ecstasy, pharmaceutical stimulants, benzodiazepines, Unisom and participants who reported >1 drug injected most often.

^3^ Among those who reported ever being tested for HCV.

^4^ Among participants who completed question (n=675 - 684); Not mutually exclusive.

^5^ Includes specialist physician, dentist, allied health service.

### Retention at twelve months follow-up

**Figure 2:**
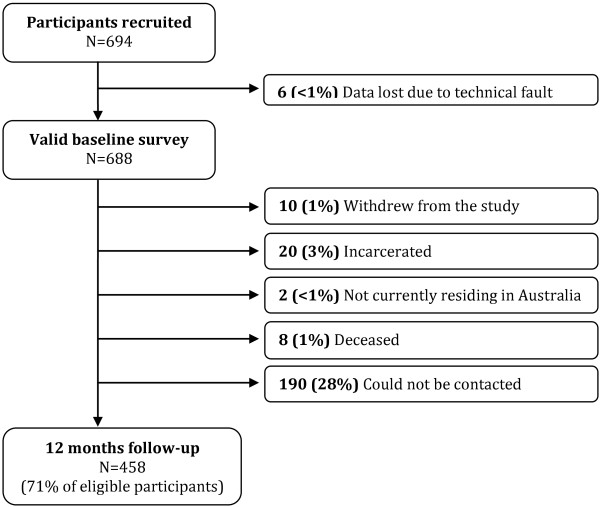
Participant flow diagram.

At twelve months follow-up, 30 participants (4%) were known to be incarcerated, to have died or to no longer be residing in Australia, and an additional 10 participants (1%) voluntarily withdrew from the study. Of the participants who were eligible for follow-up, 458 (71%) were retained in the study (Figure 2), and completed follow-up interviews a median of 357 days post-baseline (IQR: 317–435 days).

The baseline characteristics of participants who completed a 12-month follow-up interview were compared with those who did not. Independent correlates of attrition were: recruitment from Inner West or Central Melbourne, male gender, and failing to provide a telephone number or residential address at baseline (Table 3).

**Table 3:** Correlates of attrition at 12-months

**Variable**	**Followed-up**	**Not followed-up**	**Univariate**	**Multivariable**
	**N=458**	**N=230**	**OR (95% CI)**	**OR (95% CI)**
	**n (%)**	**n (%)**		
**Recruitment method**				
RDS	164 (36)	82 (36)	1	
Other	294 (64)	148 (64)	1.00 (0.72-1.40)	
**Recruitment location**				
Inner West	230 (50)	131 (57)	2.02 (1.30-3.14)**	2.10 (1.33-3.32)**
Central	111 (24)	66 (29)	2.11 (1.29-3.45)**	1.80 (1.25-2.60)**
Outer-Urban	117 (26)	33 (14)	1	1
**Sex**				
Male	285 (62)	174 (76)	1	1
Female	173 (38)	56 (24)	0.53 (0.37-0.76)**	0.56 (0.38-0.80)**
**Age**				
Median (IQR)	27.8 (24.1-29.7)	27.3 (24.6-29.4)	0.98 (0.94-1.03)	
**Aboriginal/Torres Strait Islander status**				
Yes	25 (6)	15 (7)	1	
No	433 (95)	215 (94)	1.21 (0.62-2.34)	
**Country of birth**				
Australia	373 (82)	177 (78)	1	
Other	84 (18)	51 (22)	0.78 (0.53-1.16)	
**Main income source (last month)**				
Wage or salary	36 (8)	22 (10)	1	
Government pension or benefits	392 (86)	197 (86)	0.82 (0.47-1.44)	
Other^1^	28 (6)	10 (4)	0.58 (0.24-1.43)	
**Employment status**				
Not employed	391 (86)	199 (87)	1	
Employed	66 (14)	31 (13)	0.92 (0.58-1.46)	
**Education**				
Did not complete year 10	139 (31)	91 (40)	1	
Completed year 10–11	223 (49)	94 (41)	0.64 (0.45-0.92)*	
Completed high school or higher	94 (21)	45 (20)	0.73 (0.47-1.14)	
**Current accommodation type**				
Owner-occupied	104 (23)	50 (22)	1	
Private rental	115 (25)	77 (34)	1.39 (0.89-2.17)	
Public housing	146 (32)	61 (27)	0.87 (0.55-1.36)	
No stable accommodation	90 (20)	41 (18)	0.95 (0.57-1.56)	
**Incarceration history**				
Never been in prison	194 (43)	82 (36)	1	
Incarcerated once	136 (30)	75 (33)	1.30 (0.89-1.91)	
Incarcerated two or more times	126 (28)	71 (31)	1.33 (0.90-1.97)	
**Recent arrest (last 12 months)**				
Yes	236 (52)	133 (59)	1	
No	218 (48)	94 (41)	0.77 (0.55-1.06)	
**Age at first injection**				
Median (range)	17 (11–32)	17 (8–29)	0.99 (0.94-1.03)	
**Duration of injecting career (years)**				
Median (range)	10.0 (<1-20.8)	10.3 (<1-21.2)	1.00 (0.96-1.03)	
**Ever been on OST**				
Yes	335 (73)	154 (68)	1	
No	121 (27)	74 (32)	1.33 (0.94-1.88)	
**Currently on OST**				
Yes	175 (38)	67 (29)	1	
No	281 (62)	161 (71)	1.50 (1.06-2.11)*	
**Drugs injected last month**				
Heroin only	187 (41)	92 (40)	1	
Heroin and other drugs	183 (40)	101 (44)	1.11 (0.53-2.34)	
Amphetamines only	22 (5)	12 (5)	1.47 (0.69-3.13)	
Other drugs only	48 (11)	12 (5)	0.51 (0.26-1.00)	
Did not inject last month	18 (4)	13 (6)	1.12 (0.79-1.59)	
**Telephone number provided at baseline**				
Yes	440 (96)	195 (85)	1	1
No	18 (4)	35 (15)	4.39 (2.42-7.94)**	2.90 (1.53-5.48)**
**Home address provided at baseline**				
Yes	454 (99)	212 (92)	1	1
No	4 (1)	18 (8)	9.64 (3.22-28.82)**	6.58 (2.05-21.08)**

* p<0.05, **p<0.01; Hosmer-Lemeshow test: p=0.96.

## Discussion

The MIX cohort constitutes the largest Australian community-based PWID cohort to-date, and differs from other Australian PWID cohorts in several important ways.

Firstly, our cohort is recruited from the community, and includes a large sample of out-of-treatment PWID; just over one third of our participants were prescribed OST at recruitment, compared with 51%-63% of street-based PWID and NSP-attendees interviewed in recent Victorian drug trend monitoring studies [48-50]. As such, it does not possess the selection effects associated with recruitment from a particular place, such as treatment facilities. Although PWID who regularly attend primary care centres or pharmacies to obtain pharmacotherapy treatment may be easier to retain in longitudinal studies, PWID in-treatment tend to be different to those out-of-treatment, commonly being older and further progressed in their injecting careers [34,40]. At the time of recruitment, the heroin market in Melbourne had been relatively depressed for some time [38,51], and research suggests that this reduction in heroin supply was associated with both reduced heroin injection among current injectors and reduced initiation into injecting [42,52]. This decreased the pool of newer, out-of-treatment PWID, preventing us from recruiting as large a sample of these users as hoped. Despite this, our cohort will still provide vital information about transitions into and out of drug treatment and the factors which motivate these decisions. Further, the inclusion of individuals both in and out of treatment will allow for assessments of a range of barriers to treatment as well as evaluations of the impact of treatment.

Participants in our cohort were recruited from three locations across Melbourne, where illicit drug markets and/or NSPs are located, with significant differences in socio-demographic and drug use patterns detected across sites. The Inner-West and Central areas are historically working-class and industrial; today, they include large public housing estates, and are home to significant Asian migrant populations, and more recently, refugee populations from the Horn of Africa [53-55]. Following a transition from predominantly private dealing, street-based heroin markets emerged in these areas in the mid-1990s and continue to remain active despite ongoing policing [38,53,56]. In contrast, the outer-urban recruitment site is home to a predominantly Anglo-Australian community, with manufacturing and construction the main industries [57,58]; MIX study participants from this site displayed a preference for amphetamine and pharmaceutical opiate injection, presumably reflecting limited access to heroin due to geographic distance from active heroin markets. Differences in patterns of alcohol consumption were also recorded across research sites and may reflect a number of factors including neighbourhood liquor outlet density [59] and differing cultural attitudes towards alcohol consumption. The role of the geographical environment in drug use and associated risks and harms warrants further investigation, and will be examined in future.

Rather than focusing specifically on BBV incidence or drug treatment outcomes – the main focus of previous cohorts of Australian PWID [34-37] - our study collects data on a broad range of other health outcomes, including patterns of drug injection and injecting cessation, physical and mental health, and engagement with health services. Of particular interest is the fact that although 58% of participants reported attending a GP clinic in the past month, just 17% reported recent attendance at one of the five state-funded free PWID-specific PHC clinics, despite these clinics generally being located reasonably close to participants’ residences. Further analysis is required to explore the characteristics of clients attending these services and their presenting complaints, and to understand the ways in which patterns of health service utilisation are associated with factors such as recruitment site, service availability and patterns of drug use. The use of prospective data will also enable examination of longer-term drug use and other health outcomes among PWID attending these services.

While we used a combination of RDS, traditional snowballing and street outreach to ensure that a diverse sample of PWID were included in the study, there were few significant differences across recruitment arms. While not the focus of this paper, further analysis, including the calculation of RDS-weighted population prevalence estimates, will facilitate better understanding of the usefulness of this recruitment strategy.

Despite having worked in these field sites for a number of years [60,61], and conducting formative research prior to study commencement (field-based observations and pilot interviews), the Melbourne drug market is dynamic, and unanticipated changes in both people accessing the market, and availability of different drug types did occur [62,63]. In response, a number of changes to the eligibility criteria of the study, as well as study procedures were implemented.

Firstly, we relaxed our age restriction on eligibility, which resulted in the inclusion of 95 participants aged 30–31, and 38 participants aged over 31 in the study. As such our sample is slightly older than initially hoped, with a median age of 27.6 years, making them slightly younger than participants in the Victorian cohort recruited by Crofts et al. in the early 1990s [37], but older than cohorts recruited in Sydney and Melbourne in the mid-2000s [35,36]. It has been noted that PWID in this jurisdiction are an ageing population; repeat cross-sectional surveys have indicated that the median age of NSP attendees in Victoria has increased significantly from 26 years in 1997 to 35 years in 2010 [50]. Similar increases in mean ages have been observed among PWID survey participants in Victoria’s illicit drug trends monitoring system over the past ten years [48,64]. This is likely to be due to the population of ageing PWID who initiated injecting in the 1980s and 1990s and continue to inject today, combined with decreasing numbers of young people initiating injection [42]. The median year of injecting initiation among our sample, however, was 1999 (IQR: 1996–2003), with a median delay of one year to regular injecting drug use. Thus, while a proportion of participants initiated injecting during the latter years of the heroin ‘glut’ [38], there are few participants in our study for whom drug use was already entrenched during this period, with the majority commencing regular injecting in the setting of limited heroin availability.

Our study initially aimed to recruit both primary heroin and methamphetamine injectors, as most previous Australia cohorts have been comprised mostly of heroin injectors [36,37,65] and, despite reported recent increases in crystal methamphetamine use, relatively little was known about patterns of methamphetamine injection. By the time study recruitment commenced however, recent reports of crystal methamphetamine use had again decreased [66], meaning that only a small number of primary methamphetamine injectors met the study eligibility criteria. Nonetheless, prospective data collection will enable ongoing monitoring of trends in methamphetamine use, and provide opportunities to explore potential changes in drug use and health outcomes as participants transition between different patterns of primary heroin and methamphetamine use.

While other Australian PWID cohorts have been limited by short durations of follow-up, the MIX cohort will be followed up annually for a minimum of four years (with funding for further follow-up to be sought). At 12-months’ follow-up, the retention rate was 71%, comparable to similar international studies, which had follow-up rates from 68%-83% reported over durations ranging from three months to four years [27-30,32]. Similar to other longitudinal studies of vulnerable populations, we found that the collection of detailed contact information at baseline, comprehensive follow-up procedures and an ongoing field presence that allowed researchers to build familiarity and trust with participants, were all integral in tracking respondents [67,68]; participants who did not provide complete contact details at baseline were more likely to be lost to follow-up. Importantly, while attrition was associated with male gender, those lost to follow-up were otherwise similar to participants retained in the study, thus limiting the impact of attrition bias on our findings. The long duration of follow-up, combined with future data linkage through administrative data (e.g. the Medicare system) beyond the period of face-to-face follow-up will produce rich and versatile data enabling a better understanding of the natural history of injecting drug use and patterns of morbidity and mortality (overall, as well as among particular subgroups of PWID). These data will be integral to the evaluation of health and social interventions among this group.

### Limitations

Due to ethical considerations, we were not permitted to recruit participants younger than 18 years of age, however due to a miscommunication a small number of participants aged 16 and 17 were inadvertently recruited into the study; ethics approval has been obtained to use data from these participants. It remains unclear whether this population of adolescent PWID are being targeted effectively by research or health interventions.

Given the complexities involved with street-based recruitment across multiple field sites, involving a large research team, it was not possible to monitor how many PWID were invited but declined to participate in the study. Unwillingness to consent to the provision of Medicare information may have been associated with declining to participate in the study.

As with much PWID research, our data may be limited by selection bias, and as behavioural data were self-reported, also by recall and social acceptability bias. Future data linkage and BBV testing will enable us to assess the accuracy of some self-reported variables.

## Conclusions

Although PWID can be difficult to retain in longitudinal studies, well-planned follow-up procedures and an ongoing field presence can lead to high levels of retention and minimal attrition bias. Data from the MIX cohort will allow for the exploration of the natural history of injecting drug use, and the identification of both risk and protective factors for adverse health outcomes associated with injecting drug use in Australia.

## Abbreviations

BBV: Blood borne virus; GP: General practice; MIX: Melbourne injecting drug user cohort Study; NSP: Needle and syringe exchange program; OST: Opioid substitution therapy; PDA: Personal digital assistant; PHC: Primary health care; PWID: People who inject drugs; RDS: Respondent driven sampling; STROBE: Strengthening the reporting of observational studies in Epidemiology.

## Competing interests

The authors declare that they have no competing interests.

## Authors’ contributions

DH was involved in project coordination, baseline recruitment and data collection, conducted the data analysis and led the writing of the manuscript. PH was involved in the study design, baseline recruitment and participant follow-up. RJ was responsible for data management and assisted with analysis. PD is the chief investigator on the MIX study, and a chief investigator within the Drug Policy Modelling Project. All other authors were involved in the initial study design and development, and have contributed to and approved the final manuscript.
